# Population genetic analysis of 36 Y-chromosomal STRs yields comprehensive insights into the forensic features and phylogenetic relationship of Chinese Tai-Kadai-speaking Bouyei

**DOI:** 10.1371/journal.pone.0224601

**Published:** 2019-11-08

**Authors:** Ya Luo, Yan Wu, Enfang Qian, Qian Wang, Qiyan Wang, Hongling Zhang, Xiaojuan Wang, Han Zhang, Meiqing Yang, Jingyan Ji, Zheng Ren, Ying Zhang, Jing Tang, Jiang Huang

**Affiliations:** 1 Department of Forensic Medicine, Guizhou Medical University, Guiyang, Guizhou, China; 2 Guiyang Judicial Expertise Center of Public Security, Guiyang, Guizhou, China; National Cheng Kung University, TAIWAN

## Abstract

Male-specifically inherited Y-STRs, harboring the features of haploidy and lack of crossing over, have gained considerable attention in population genetics and forensic investigations. Goldeneye® Y-PLUS kit was a recently developed amplification system focused on the genetic diversity of 36 Y-chromosomal short tandem repeats (Y-STRs) in East Asians. However, no population data and corresponding forensic features were reported in China. Here, 36 Y-STRs were first genotyped in 400 unrelated healthy Tai-Kadai-speaking Bouyei male individuals. A total of 371 alleles and 396 haplotypes could be detected, and the allelic frequencies ranged from 0.0025 to 0.9875. The haplotype diversity, random match probability and discrimination capacity values were 0.9999, 0.0026 and 0.9900, respectively. The gene diversity (GD) of 36 Y-STR loci in the studied group ranged from 0.0248 (DYS645) to 0.9601 (DYS385a/b). Population comparisons between the Guizhou Bouyei and 80 reference groups were performed via the AMOVA, MDS, and phylogenetic relationship reconstruction. The results showed that the population stratification was almost consistent with the geographic distribution and language-family, both among Chinese and worldwide ethnic groups. Our newly genotyped Bouyei samples show a close affinity with other Tai-Kadai-speaking groups in China and Southeast Asia. Our data may provide useful information for paternal lineage in the forensic application and population genetics, as well as evidence for archaeological and historical research.

## Introduction

Since the Y-chromosomal short tandem repeats (Y-STRs) were discovered in 1992, they have been regarded as valuable markers in forensic analysis, population genetics, and evolutionary studies [1, 2], such as paternal lineage searching for the suspect, kinship analysis, research of involving population lineage and human migration[3]. In recent decades, many commercially available Y-STR kits have been studied[4, 5], as well as the population genetic data used to set up the Y-STR reference databases[6]. Goldeneye® Y-PLUS kit (Peoplespot Technology Ltd., Beijing, China) was recently developed and validated next-generation amplification Y-STR amplification system, which included 27 Y-STRs included in the Y-Filer kit and other 9 new focused loci, including DYS19, DYS460, DYS389 I, DYS389 II, DYS390, DYS391, DYS392, DYS393, DYS437, DYS438, DYS439, DYS448, Y-GATA H4, DYS449, DYS456, DYS458, DYS481, DYS533, DYS570, DYS627, DYS635, DYS576, DYS388, DYS549, DYS444, DYS643, DYS447, DYS557, DYS596, DYS593 and DYS645, DYS518, and four multiple copy loci, namely DYS385, DYF387S1, DYS527 and DYF404S1. Additionally, DYF387S1, DYF404S1, DYS449, DYS570, DYS576, DYS518 and DYS627 were reported as rapidly mutating (RM) Y-STR[7].

China is a multi-ethnic country with 55 minorities. The Bouyei, which has a population of approximately 2.87 million in the 2010 census, is one of the most widely distributed ethnic groups in southwestern China. Many Bouyei people live in Guizhou province, accounting for 97% of the total Bouyei population[8, 9]. The Bouyei people have their own language belonging to the Tai-Kadai family, which is similar to Zhuang, Dai, Dong, Li and Thai, and the Bouyei language has its own characters/writing system. Because of the geographical features and their unique ethnic language and culture, the Guizhou Bouyei rarely intermarried with other ethnic groups and relatively isolated from other populations. Thus, it is necessary in order to explore the origin and the Chinese Bouyei people, and the genetic relationship and population stratification with other ethnic groups as well.

Many published population genetic data Guizhou Bouyei were focused on autosomal-STRs[10], X-Chromosomal-STRs[11, 12], mitochondrial genome genetic markers and 23 and even fewer Y-chromosomal markers[13–15], which may be not enough for forensic or Anthropological purpose. Thus, in this study, we obtained the haplotype data from 400 Guizhou Bouyei unrelated male individuals using the Goldeneye® Y-PLUS kit. Furthermore, we combined data of Bouyei and other 100 populations available in the published database, which were divided by geographical distribution, ethnic administrative and national boundaries, to analyze genetic relationships between different ethnic groups and population stratification. Our research can enrich the genetic database of Bouyei ethnic groups for forensic, population genetic, and national evolutionary purposes, and reveal the genetic characteristics of this Chinese minority, and the genetic relationship between other reference populations.

## Materials and methods

### Subjects and sample collection

**Fig 1:**
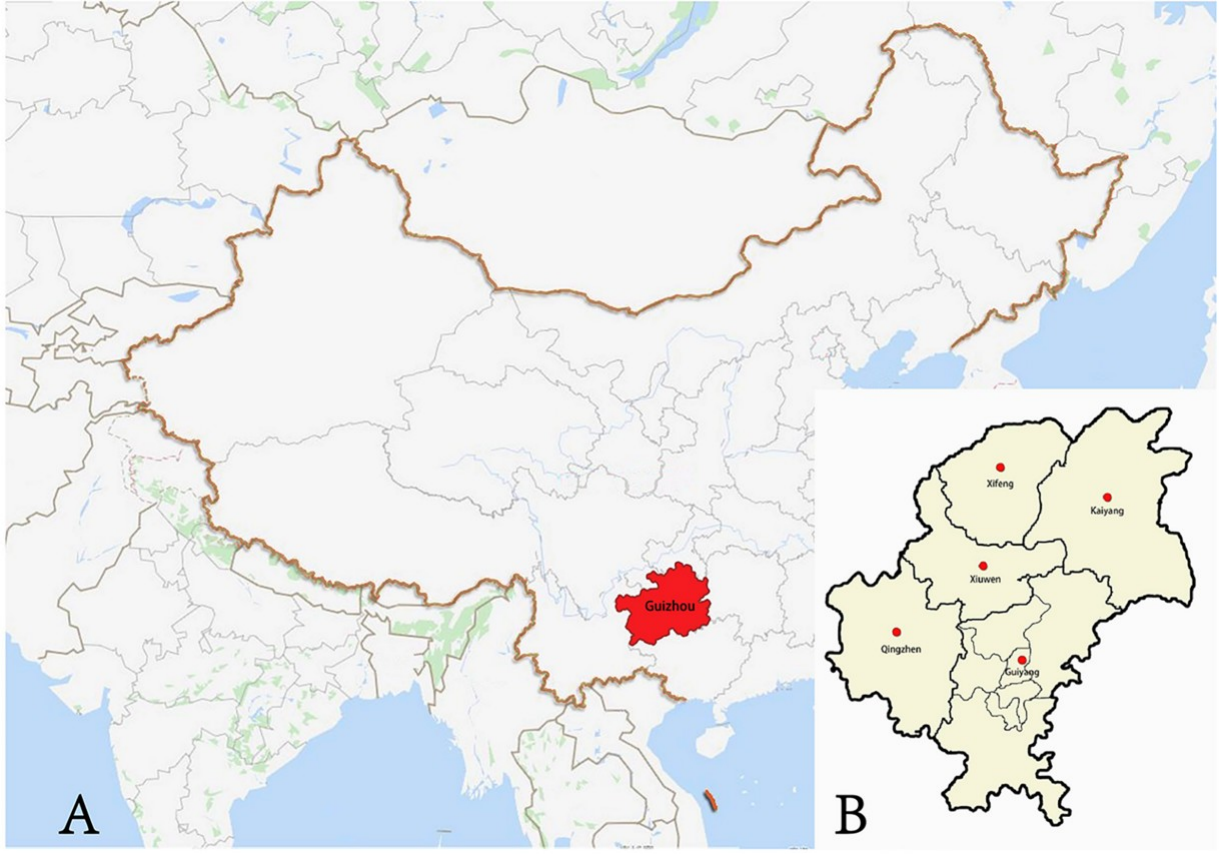
Geographic locations of Guizhou Bouyei in present study.

Peripheral blood samples were collected from a total of 400 unrelated healthy Bouyei people residing in Guiyang, Guizhou province (Southwest China). The geographic distribution of the studied populations is shown in Fig 1. All the participant consents have been obtained by written form in the informed consent. The ancestors of all subjects must live in the present region for at least three generations. We conducted this study strictly followed the human and ethical research principles, which was approved by the Medical Ethics Committee of Guizhou Medical University. And informed consent was obtained from all the participating individuals.

### Multiplex amplification and genotyping

Thirty-six Y-STR loci were co-amplified in one multiplex PCR reaction on a GeneAmp PCR System 9700 (Thermo Fisher Scientific, Wilmington, DE, USA) from the FTA card, using Goldeneye® Y-PLUS kit (Peoplespot Technology Ltd., Beijing, China) based on the manufacturer’s instructions. DNA was amplified using 10μL reaction volume, which contained 2ul reaction mix, 2μL primers, 1μL A-Taq DNA polymerase and 6μL sdH2O. PCR conditions were 95°C for 2 min, followed by 30 cycles of 94°Cfor 1 min, 60°Cfor 45s, and 72°Cfor 45s, and a final extension at 60°C for 45min. PCR products were separated on the ABI 3730 Genetic Analyzer (Thermo Fisher Scientific, Wilmington, DE, USA) with the POP-7 polymer. The electrophoretic sampling mixture included 1 μL amplified product 10 μL Hi-Di formamide and 1 μL ORG500 size standard. Standard DNA templet 9947A was analyzed for positive control, and sdH2O for negative control as well. Allele nomenclature was conducted using the GeneMapper ID-X v.1.4 software.

### Statistical analysis

Allele frequencies of 36 Y-STR loci and haplotype frequencies were calculated using the direct counting method. Forensic statistical parameters of gene diversity (GD) and haplotype diversity (HD) were calculated using the Nei’s formula[16]: HD = (n/n − 1) (1 − ∑ Pi^2^), where n was the total number of samples and Pi was the frequency of the ith haplotype; GD = (n/n − 1) (1 − ∑ Pi^2^), where n was the total number of samples and Pi was the frequency of the ith allele. Haplotype match probability (HMP) was calculated according to HMP = ∑ Pi^2^, where Pi was the frequency of the haplotype. Discrimination capacity (DC) was calculated based on the formula: DC = k/Σ(Pi×n), where k was the number of haplotypes, Pi was the frequency of its haplotype, n was the total number of individuals.

Comprehensive populations comparisons at different scales based on Y-chromosomal STR haplotype data were performed to investigate genetic similarities and differences between our studied population and reference populations. Pairwise Rst was computed based on 27 Y-STR loci (Y-filer Plus set) between Guizhou Bouyei and reference populations extracted from the Y Chromosome Haplotype Reference database YHRD[17], including 9 Han populations (Guangxi Han, Henan Han[18, 19], Jiangxi Han, Nantong Han[20], Shanghai Han[21], Zhejiang Han). 18 Chinese minority ethnic groups (Inner Mongolia Daur, Gansu Dongxiang[22], Guizhou Gelao, Gansu Hui[23], Xinjiang Hui, Xinjiang Kazakh, Yanbian Korean, Hainan Li[24, 25], Hainan Lingao[26], Guizhou Miao, Gansu Tibetan, Qinghai Tibetan[27], Hubei Tujia, Xinjiang Uighur, Guizhou, Sichuan and Yunnan Yi[28], Guangxi Zhuang[29]), 36 worldwide populations (Lithuania Lithuanian[30], Poland Polish, Russian Federation Russian, Denmark Danish, Berlin-Brandenburg German[3], Ljubljana Slovenia Slovenian, Budapest Hungary Hungarian, Libya Arab Jewish[31], Algeria Mozabite Berber[31], Morocco[31], Egypt [31], Djibouti Somali Afar, Somalia Somali, Ethiopia[32], Macedonia Macedonian, Italy Italian[33], Bergamo Italy Italian, Switzerland Swiss[34], Ireland Irish[35], Madrid, Spain Spanish, Singapore Malay, Laos Laotian, Thailand Thai, Guangdong Han[36], Dezhou Han, Cebu Philippines Cebuano, South Korea Korean, Daejeon South Korea Korean[37], Aomori Japan Japanese, Ehime, Japan Japanese, Gunma Japan Japanese, Hyogo Japan Japanese, Okayama Japan Japanese, Okinawa Japan Japanese, Eritrea Saho, 17 meta-populations (Belgium, Denmark, Germany, India, Kenya, Russian Federation, Singapore, Somalia, South Korea, Spain, United States (S1 Table). Pairwise Rst genetic distances were calculated by analysis of molecular variance (AMOVA) and visualized in multidimensional scaling (MDS) plot using the AMOVA&MDS tool on the YHRD. Finally, A neighbor-joining (NJ) phylogenetic tree was constructed based on the Rst matrix using the MEGA 6.0[38].

### Quality control

This study strictly followed ISFG recommendations on the analysis of the DNA polymorphisms and nomenclature[39] and guidelines for publication of population data[40]. Our lab also has accredited with the China National Accreditation Service for Conformity Assessment (CNAS). Our data has been submitted to the YHRD database with the accession number of YA004543.

## Results

### Y-chromosomal genetic diversity in Guizhou Bouyei

We genotyped 36 Y-STRs in a total of 400 Guizhou Bouyei individuals successfully (S2 Table), and a total of 396 different haplotypes were observed among 400 individuals, of which 392 were unique. The HD and DC were found to be 0.9999 and 0.9900, respectively. HMP was 0.0026. Forensic parameters, including allele frequencies and GD, were listed in S3 Table. The corresponding allelic frequencies varied from 0.0025 to 0.9875, and the GD ranged from 0.0248 (DYS645) to 0.9601 (DYS385a/b). All studied loci get GD values higher than 0.5 except for DYS645 (0.0248), DYS438 (0.3935), DYS391 (0.4089), DYS596 (0.4885), DYS437 (0.4890).

### Genetic differentiation along national or continental geographical divisions

**Fig 2:**
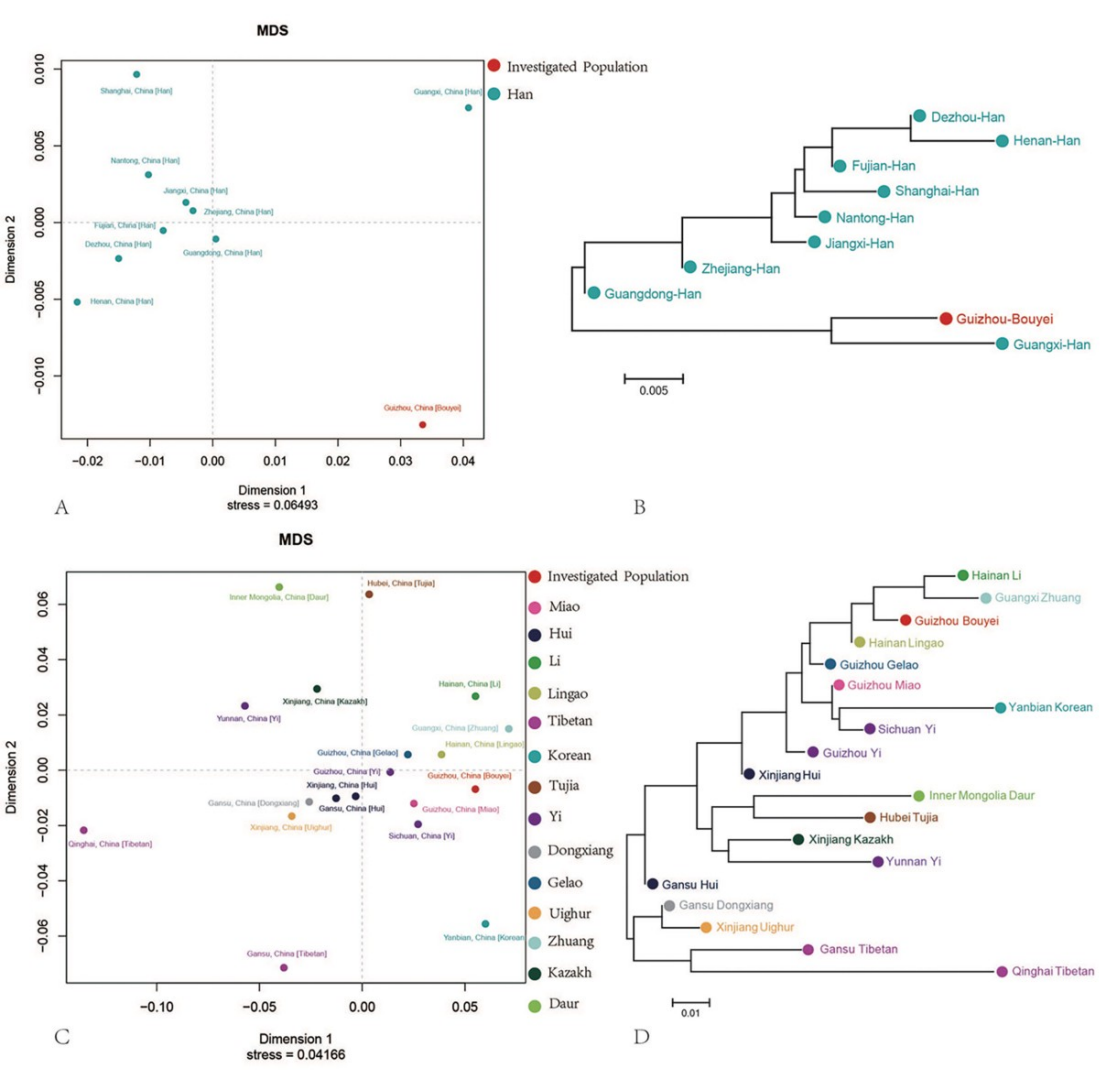
Genetic similarities and differences among our target and reference populations along administrative or national boundaries. (A) The Neighbor-Joining tree shows the genetic affinity and divergence among 36 reference populations. (B) Multidimensional Scaling plots of our studied population and 17 Meta-populations based on Y-chromosomal haplotypes. (C) Phylogenetic relationship between 17 Meta-populations and our investigated population.

To reveal the partial population substructure between large-scale geographic divisions, Rst values between our studied subject and 36 populations with 8623 haplotypes from Asia, Europe and Africa were calculated based on 27 Y-STRs. As shown in S4 Table, the pairwise Rst values range from 0.0129 to 0.4392 for Guizhou Bouyei, and our target was first clustered with Laos Laotian and Thailand Thai in the phylogenetic relation reconstruction tree. For all of the populations included, four clear genetic affinity clusters could be identified: East Asian cluster, European cluster, Southeast Asian cluster, and African cluster (Fig 2A). The Bouyei was firstly clustered with two Southeast Asian populations Thai and Laotian instead of Chinese Han.

Subsequently, to further explore the partial population substructure between large-scale geographic divisions, we calculated pairwise Rst values between our subject and 17 meta-populations (combination on the basis of national or local boundaries) with 9838 haplotypes from Asia, Europe, Africa and America, as showed in S5 Table. Multidimensional scaling plot in Fig 2B showed a genetic cluster consisting of our subject, South Korea, Japan, Thailand and Singapore were isolated and located in the upper left corner, Italy, Belgium, Germany, Denmark, Spain, Russian Federation in the lower-left corner, and Somalia, Kenya, Libya, Morocco scattered in the right. Guizhou Bouyei was also firstly clustered with Thailand and subsequently clustered with Japan and South Korea, finally clustered with Singapore converged one clade (Fig 2C), showing a geographic affinity of Bouyei population with other Asian groups.

### Genetic differentiation along with mainland Chinese administrative and ethnic divisions

**Fig 3:**
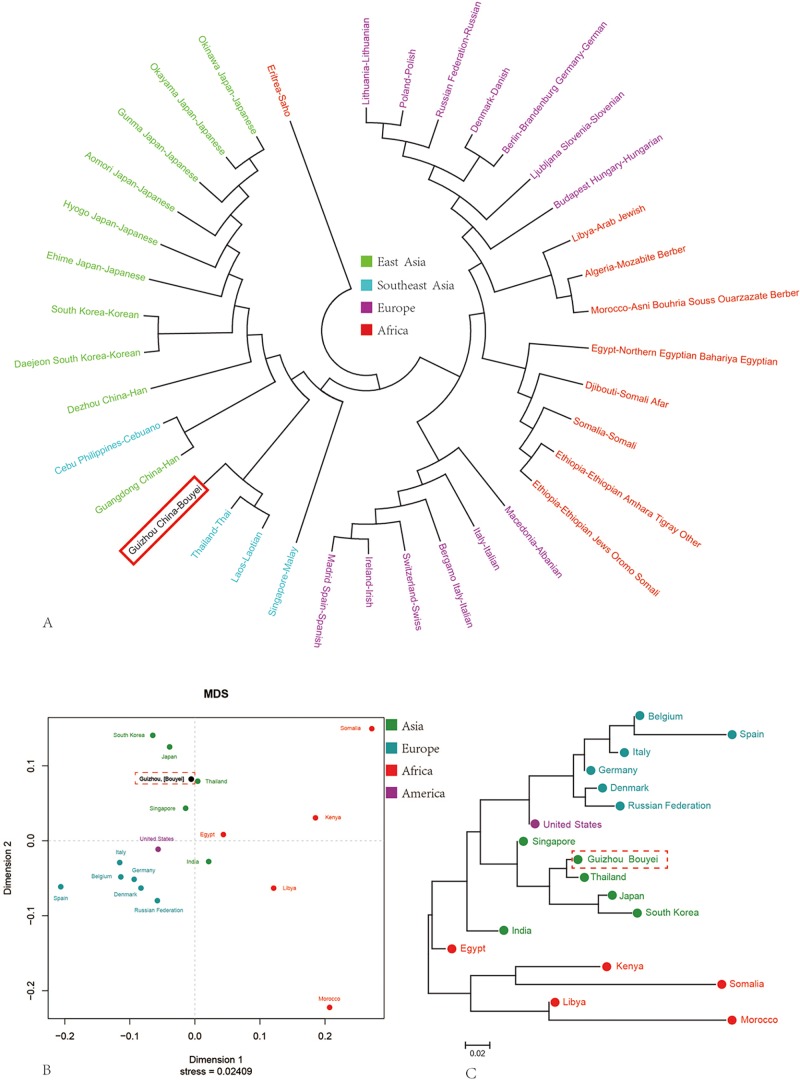
Genetic relationships between Guizhou Bouyei and reference populations defined by ethnic origin and administrative divisions. (A) Multidimensional scaling plots show the genetic correlation between Guizhou Bouyei and 9 Han Chinese populations. (B) Phylogenetic relationship between our target and 9 Han Chinese populations. (C) Multidimensional scaling plots show the genetic differentiation between the studied population and 18 Chinese minority ethnicities. (D) The Neighbor-Joining tree was constructed based on Rst genetic distance matrix among 19 populations.

9303 Y-STR haplotypes from 9 Han Chinese populations were used to investigate the degree of differentiation between our studied subject and 9 Han Chinese populations via analysis of molecular variance (AMOVA), S6 Table listed the Rst values among 10 groups and shows that the largest genetic distance is observed between Guizhou Bouyei and Henan Han (Rst = 0.0705), while the closest genetic relationships were with the Guangxi Han (0.023). As demonstrated in the MDS plot Fig 3A, the Guizhou Bouyei was relatively isolated from other populations. According to phylogenetic tree Fig 3B, Guizhou Bouyei and Guangxi Han converged closely and formed one clade, and the other was formed by the remaining Han populations.

8010 Y-STR haplotypes from 18 populations were employed to calculate the pairwise Rst values as showed in S7 Table. The largest genetic distance was detected between Guizhou Bouyei and Qinghai Tibetan (Rst = 0.2172), and the closest genetic relationships with Hainan Lingao (0.02). MDS plot in Fig 3C revealed substantial genetic distances among Chinese ethnicities, especially between Hubei Tujia, Inner Mongolia Daur, Qinghai Tibetan, Gansu Tibetan and Yanbian Korean and other Chinese populations. Neighbor-Joining tree plot in Fig 3D showed two separate clusters, one of which comprised northwestern Chinese ethnicities (Qinghai Tibetan, Gansu Tibetan, Xinjiang Uighur and Gansu Dongxiang), and the other consisted of the remaining 14 populations. Our studied Bouyei was first grouped with Guangxi Zhuang and Hainan Li.

## Discussions

In our study, we used the Goldeneye® Y-Plus kit including 36 loci to investigate the Guiyang Bouyei (Guizhou, China) population, which contained all markers of previous commercial kits (Minimal haplotype, PowerPlex^®^ Y, AmpFlSTR® Yfiler, PowerPlex® Y23 and AmpFlSTR® Yfiler Plus), and other two RM Y-STRs (DYF404S1 and DYS449) and one multi-copy loci (DYS527). Thus, the DC value of our system (0.9900) was higher than the 27 Y-STRs (Yfiler Plus) (0.9825), demonstrating that it was highly informative and polymorphic, and exhibited great efficiency in Guiyang Bouyei populations investigated in this study. However, there were still five loci with a low level of polymorphism, including DYS645 (0.0248), DYS438 (0.3935), DYS391 (0.4089), DYS596 (0.4885), DYS437 (0.4890), which seemed not to be suitable for forensic purpose in this population.

Y-chromosomal markers have been widely used for studying the origin of modern humans, inferring the male genetic genealogy evolution, and dissecting the population stratification for constructing regional effective forensic reference database. Although Chinese male genetic landscape was revisited by 38,000 17-Y-STR haplotypes study[8], the analysis of population stratification and genetic relationships among Chinese Han and minorities based on only 17 loci may not be accurate enough. Chen at al [41] also investigated the genetic diversity of 98 Qiannan Bouyei, 101 Zunyi Han and 109 Qiandongnan Miao individuals based on 23 Y-STRs loci, all of which located in Guizhou province, but more attention should be still paid in the forensic practices and population genetic applications due to the relatively small sample size.

Thus, in this study, we reported the 27 Y-STRs from 400 Guizhou Bouyei samples to shed more light on the genetic relationships of Chinese national and worldwide populations including 9 Han population, 18 minority populations in China, and 36 Asian, European and African populations. Our results demonstrated that Guiyang Bouyei was genetically distant with all Han populations. Among them, Guangxi Han had a closest genetic affinity with Bouyei, which was consistent with the geographic distribution. For all the minorities, Guizhou Bouyei has a close genetic affinity with Guangxi Zhuang, Hainan Lingao and Guizhou Miao, while it was genetically distant from Tibetan in China. This situation was also consistent with the language family divisions. For example, Guizhou Bouyei, Guangxi Zhuang, Hainan Lingao and Hannan Li were all Tai-Kadai-speaking populations. It was also consistent with the results reported by Zhang et al. [42] according to autosomal InDel analysis. However, there were still some exceptions, such as Yanbian Korean and Sichuan Yi, which belonging to isolated Korean and Sino-Tibetan language-family, respectively, and were distributed in northeast and southwest of China, respectively, and the same was true for the Inner Mongolia Daur (Altaic-speaking) and Hubei Tujia (Sino-Tibetan-speaking). This should further develop the study of these ethnic groups by other kinds of genetic markers.

For estimating the stratification of worldwide populations, we calculate Rst values between our subject and 36 ethnic groups and 17 meta-populations from Asia, Europe, Africa and America, and found that they were clustered as each continent they located, demonstrating a strong association between genetic distance and geographical distribution. However, our subject clustered with Thai firstly, followed by Laotian, instead of Chinese Han, which is consistent with language classification since Bouyei, Laos and Thai all speak Tai-Kadai languages. For the polygenetic tree based on meta-populations across the world, each group clustered according to their locations strictly, and the American populations firstly incorporated into the European branch, as well as the Indians to the East Asian branch. Considering that the public data Y-STRs in Bouyei, especially from other regions in China, is rare currently, it is difficult to explore the population stratification within this population at paternal lineage. Thus, investigating more Y-STR data to improve the various Bouyei population database should be taken into consideration.

## Conclusion

Here, we reported a detailed 36 Y-STR loci data of Guizhou Bouyei population, contributing to enlarge the knowledge on the genetic landscape of China. The Y-STR haplotypes are highly polymorphic (HD: 0.9999) and have a high power of discrimination (DC: 0.9900). Additionally, the genetic relationships between Guizhou Bouyei and 61 ethnic populations and 17 meta-groups based on 27 Y-STRs showed that the population stratification was almost consistent with geographic distribution and language-family, both among Chinese and worldwide ethnic groups.

## Supporting information

S1 Table The detailed information of included reference populations. (XLSX)

S2 Table The haplotype distributions for the 36 Y-STR loci in Bouyei group (n = 400). (XLSX)

S3 Table Genetic diversities (GD) and allelic frequencies for the 36 Y-STR loci in Bouyei group (n = 400). (XLSX)

S4 Table The pairwise genetic distances between Guizhou Bouyei and 36 reference wold populations. (XLSX)

S5 Table The pairwise genetic distances between Guizhou Bouyei and 17 Meta-populations. (XLSX)

S6 Table The pairwise genetic distances between Guizhou Bouyei and 9 reference Han Chinese populations. (XLSX)

S7 Table The pairwise genetic distances between Guizhou Bouyei and 18 reference Chinese minority populations. (XLSX)
